# Respiratory virus modulation of host nucleocytoplasmic transport; target for therapeutic intervention?

**DOI:** 10.3389/fmicb.2015.00848

**Published:** 2015-08-14

**Authors:** Leon Caly, Reena Ghildyal, David A. Jans

**Affiliations:** ^1^Nuclear Signaling Laboratory, Department of Biochemistry and Molecular Biology, Monash University, Clayton, VICAustralia; ^2^Faculty of ESTeM, University of Canberra, Bruce, ACTAustralia

**Keywords:** rhinovirus, nuclear transport, importins, anti-viral strategies, influenza, respiratory syncytial viruses, exportins

## Abstract

The respiratory diseases caused by rhinovirus, respiratory syncytial virus, and influenza virus represent a large social and financial burden on healthcare worldwide. Although all three viruses have distinctly unique properties in terms of infection and replication, they share the ability to exploit/manipulate the host-cell nucleocytoplasmic transport system in order to replicate effectively and efficiently. This review outlines the various ways in which infection by these viruses impacts on the host nucleocytoplasmic transport system, and examples where inhibition thereof in turn decreases viral replication. The highly conserved nature of the nucleocytoplasmic transport system and the viral proteins that interact with it make this virus–host interface a prime candidate for the development of specific antiviral therapeutics in the future.

## Introduction: the Global Health Problem

Viral respiratory disease (VRD) results in the hospitalization and deaths each year of millions of people worldwide, representing a large social and financial burden on healthcare globally. Although 100s of viruses can potentially cause VRD, the main causative agents are, Influenza virus, RSV, and HRV. Influenza virus, an orthomyxovirus, is responsible for an estimated 3–5 million cases of severe illness and 250–500 thousand deaths worldwide per year, with an economic impact of $87.1 billion in the US alone (Molinari et al., 2007). During an epidemic/pandemic year, such as the recent 2009 H1N1 outbreak, these figures can rise dramatically; an estimated 42–86 million cases of infection were reported in 2009 (Centers for Disease Control and Prevention, 2010).

The pneumovirus RSV is the single greatest cause of lower respiratory tract illness (LRTI) and bronchiolitis in infants and the elderly, with an estimated 64 million infectious cases and 160,000–600,000 deaths recorded worldwide each winter (Falsey et al., 1995; Law et al., 2002; Simoes, 2008; WHO, 2009; Krilov, 2011). RSV related disease represents a cost of US$2.4 billion in the US alone (Tran et al., 2013). The Picornavirus HRV, is the primary causative agent of the “common cold,” resulting in upper respiratory tract infection (URTI) that is generally cleared; although severe complications can arise in vulnerable individuals including the elderly, but especially those with underlying respiratory conditions such as asthma (Nicholson et al., 1993; Costa et al., 2014), where HRV has been identified as the causative agent in 50–85% of virally induced asthma hospitalization cases, costing billions of dollars (Costa et al., 2014).

Influenza virus, RSV, and HRV alone represent a huge burden of disease and economic strain worldwide. Unlike influenza virus, where a seasonal vaccine is available, there are currently no efficacious vaccines or treatments for either RSV (Bawage et al., 2013) or HRV (Jacobs et al., 2013). Research over the past decade indicates that many cytoplasmically replicating RNA viruses, such as RSV and HRV, utilize and/or manipulate the host-cell nuclear transport machinery to their benefit, either by transporting specific viral proteins into the nucleus to modulate cellular function/minimize the host antiviral response, or by inhibiting host nuclear transport itself and thereby dampening innate immune responses (Alvisi et al., 2007; Fulcher and Jans, 2011). In the case of influenza virus, nuclear transport, and localization of key viral proteins is required for vRNA replication to occur, which is subsequently exported from the nucleus (Whittaker et al., 1996; Neumann et al., 1997; Samji, 2009; Huet et al., 2010). The importance of the host-cell nucleocytoplasmic transport machinery to viral infection makes it a therapeutic target of great potential for development of anti-viral agents in the future (Perwitasari et al., 2014).

## Nuclear Transport

### Gaining Access to the Nucleus

The nucleus is a specialized compartment within eukaryotic cells where the genetic information is contained, surrounded by the lipid double membrane structure of the NE representing the boundary between the genome and the cytoplasm (Dingwall and Laskey, 1992). Specific mechanisms are required to effect the transport of proteins, mRNA, and protein–RNA complexes between the cytoplasm and the nucleus in a controlled and regulated manner (Jans and Hubner, 1996). In order to permit the necessary passage of proteins and mRNA into and out of the nucleus, the NE is perforated by a series of NPCs through which all transport into and out of the nucleus occurs. These channels comprise up to 50 different nup proteins (8–32 copies of each; Rout et al., 2000) resulting in a super-protein-complex of around 125 MDa (Reichelt et al., 1990). Specific nups harbor hydrophobic (Phe-Gly or FG) repeat sequences, which are believed to function as transient binding sites for complexes passing through the NE. The NPC acts as a molecular sieve, enabling the passage of molecules <50 kDa in molecular weight into or out of the nucleus by passive diffusion (Talcott and Moore, 1999). Larger molecules can only be transported through the NPC in an active energy-dependent mechanism requiring specific targeting signals, NLS and NES, which mediate transport into and out of the nucleus, respectively.

**FIGURE 1:**
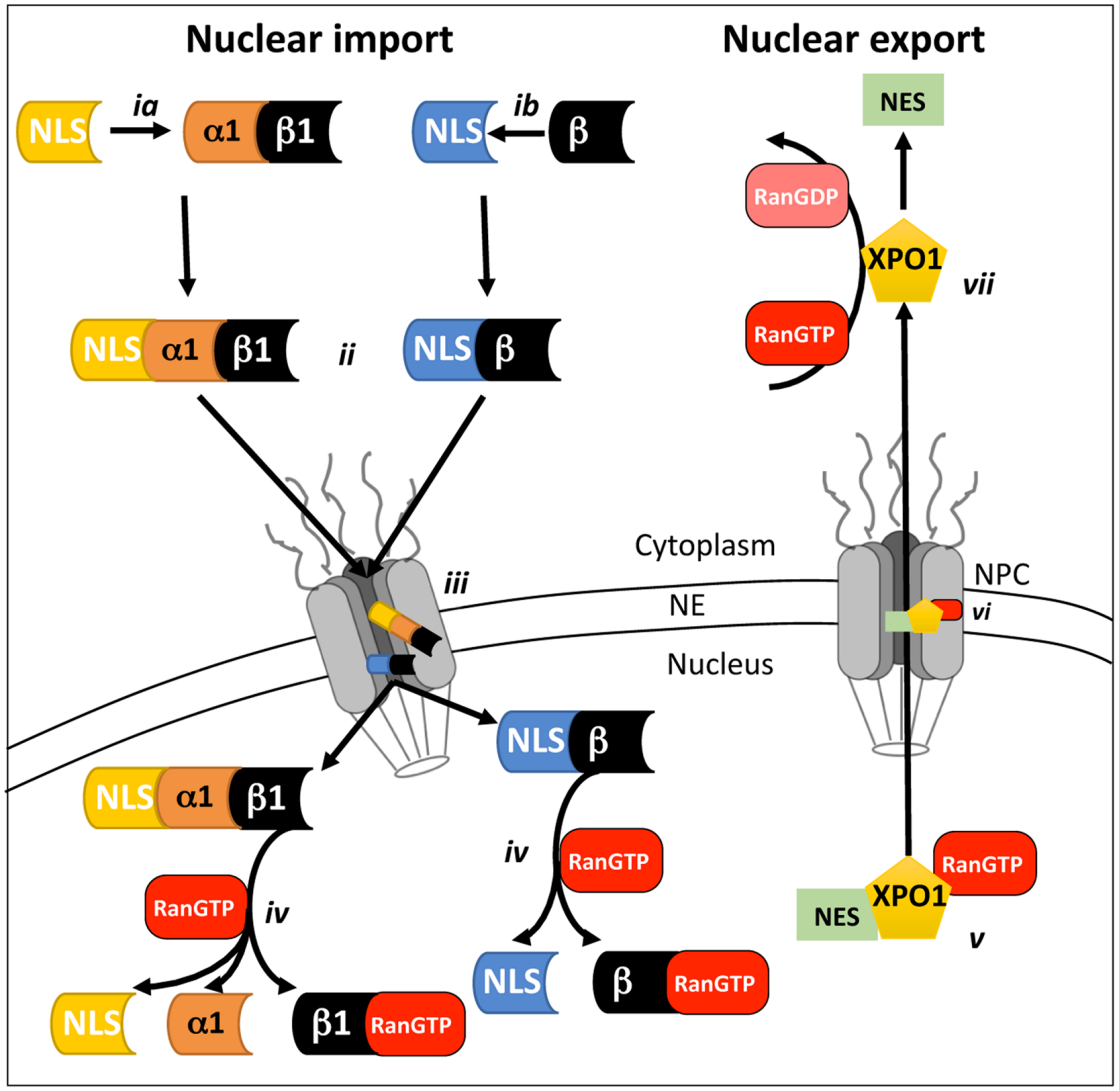
**Schematic representation of mammalian cell nucleocytoplasmic transport**. Nuclear transport across the NE requires the recognition and binding of NLS-containing cargo proteins by either the IMPα/β1 heterodimer **(ia)** or IMPβ homologues alone **(ib)** on the cytoplasmic side of the NPC. Once bound **(ii)**, the transport complex is believed to dock to the cytoplasmic side of the NPC and then move through the NPC **(iii)** via a series of transient interactions between IMPβ and the nups that comprise the NPC. Once within the nucleus, binding of RanGTP **(iv)** to Impβ causes dissociation of the transport complexes and release of the cargo to perform its nuclear function. Nuclear export **(v)** requires the recognition of a nuclear export sequence (NES)-containing cargoes by an XPO such as XPO1 in complex with RanGTP. The trimeric RanGTP/XPO1/cargo complex passes through the NPC via a series of transient interactions **(vi)** with nups within the NPC. Once within the cytoplasm, hydrolysis of RanGTP to RanGDP **(vii)** results in dissociation of the XPO1/cargo complex.

Signal-dependent protein nuclear import (see **Figure 1**) is mediated by members of the IMP superfamily of transporters, of which various α and β subtypes exist that recognize and bind to specific and highly conserved NLSs on their respective cargo proteins (Conti et al., 1998); generally through either the classical IMPα/β1 heterodimer (see **Figure 1ia**) or one of the IMPβs alone (**Figure 1ib**; Jans and Hubner, 1996; Alvisi et al., 2007; Fulcher and Jans, 2011). The NLS-cargo/IMP complex then docks to nups at the cytoplasmic side of the NPC (**Figure 1ii**), before translocating through the pore via transient and sequential interactions between IMPβ and the nups (**Figure 1iii**; Bednenko et al., 2003). Once within the nucleus, RanGTP binds to IMPβ (**Figure 1iv**) resulting in NLS-cargo release (Stewart, 2007). The nuclear IMPs are then recycled to the cytoplasm where they are available for subsequent rounds of import (Kutay et al., 1997). In analogous fashion to import, the nuclear export of NES-containing proteins is mediated by the XPO family of homologs of IMPβ1, of which XPO1 is the best-characterized (Hutten and Kehlenbach, 2007). Briefly, RanGTP binding to the XPO (**Figure 1v**) is required to allow potential cargo proteins to bind. The RanGTP/XPO/cargo trimeric complex then passes through the NPC (**Figure 1vi**) to the cytoplasm via sequential interactions between the XPO and nups of the NPC. Once in the cytoplasm, hydrolysis of RanGTP to RanGDP (**Figure 1vii**) effects release of the NES containing cargo protein into the cytoplasm.

### Nucleocytoplasmic Transport and the Innate Immune Response to Viral Infection

Viral respiratory tract infections represent one of the most common types of infectious disease(s) encountered, so it is imperative that the host innate immune response is able to identify and eliminate these threats efficiently. During viral infection, the family of TLRs (Thompson and Locarnini, 2007) in concert with the cellular helicases RIG-I and MDA-5 recognize different viral by-products, or PAMPs, which initiates a cascade of events resulting in the activation of transcription factors such as IFN response factor 3 (IRF3; Hiscott, 2007), nuclear factor κB (NF-κB; Fagerlund et al., 2005) or activating protein 1 (AP1). These transcription factors are transported to the nucleus through their interaction with specific IMPs (Torgerson et al., 1998), resulting in the initiation of IFN-β transcription. Newly translated IFN-β protein is then secreted from the cell to work in an autocrine and paracrine matter, whereby binding to the IFN-α/β receptor leads to a secondary cascade of events involving multiple proteins and transcription factors such as the STAT (signal transducer and activator of transcription) proteins. These proteins are transported to the nucleus in an IMP mediated manner where they interact with IFN-sensitive response elements (ISRE), activating the transcription of numerous anti-viral IFN stimulated genes (ISGs). Clearly, it is imperative that host-cell nuclear import remains functional during a viral infection for a concerted immune response to be initiated. With regulated host-cell nuclear transport playing such a critical role in the innate immune response, this system is therefore ripe for attenuation/modulation by infectious pathogens.

## Viral Replication and Interaction(s) with Host-Cell Nucleocytoplasmic Transport

### Human Rhinovirus (HRV)

Human rhinovirus, a member of the *picornaviridae* family, is a non-enveloped icosahedral virus that at its core possesses a positive sense single-stranded RNA (+ssRNA) genome that encodes 11 proteins initially expressed as a single polyprotein. To date there have been 156 different HRV serotypes identified (divided into serotypes A–C based on the phylogenetic relationship of the respective VP1 and VP2/4 genes; McIntyre et al., 2013). Current understanding is that the majority of HRV A/B serotypes can bind specifically to ICAM-1 on the host cell surface (Greve et al., 1989), with approximately 10% utilizing the LDLR (Hofer et al., 1994). A very small subset appear able to utilize the DAF protein (Blomqvist et al., 2002) to bind target cells, while the Cadherin-related family member 3 (CDHR3) has only recently been identified as the cell surface receptor used by HRV-C serotypes (Bochkov et al., 2015). Viral binding and attachment to the host-cell has traditionally been viewed as a viable target for drug development, but the fact that at least four different cell-surface receptors are used by HRV serotypes means that a pan-serotype inhibitor of HRV binding is unlikely to be a realistic possibility in the near future. Rhinovirus infection is initiated by inhalation of HRV into the nasal passage whereby the virions make their way to the rear of the nose where they bind one of the respective cell surface receptors. Upon binding the virions are internalized by either clathrin-dependent endocytosis or macropinocytosis (reviewed in Fuchs and Blaas, 2010), after which viral uncoating occurs, and the +ssRNA genome is released into the cytoplasm where it is translated on entry to produce a single polyprotein. The polyprotein undergoes self-proteolysis during translation by the viral proteases 2A and 3C (Skern et al., 1985; Cordingley et al., 1990) to generate the structural (VP1, VP2, VP3, VP4) and non-structural (2A, 2B, 2C, 3A, 3B, 3C, 3D) proteins required for virion assembly, meaning that the full-length product is rarely observed.

**FIGURE 2:**
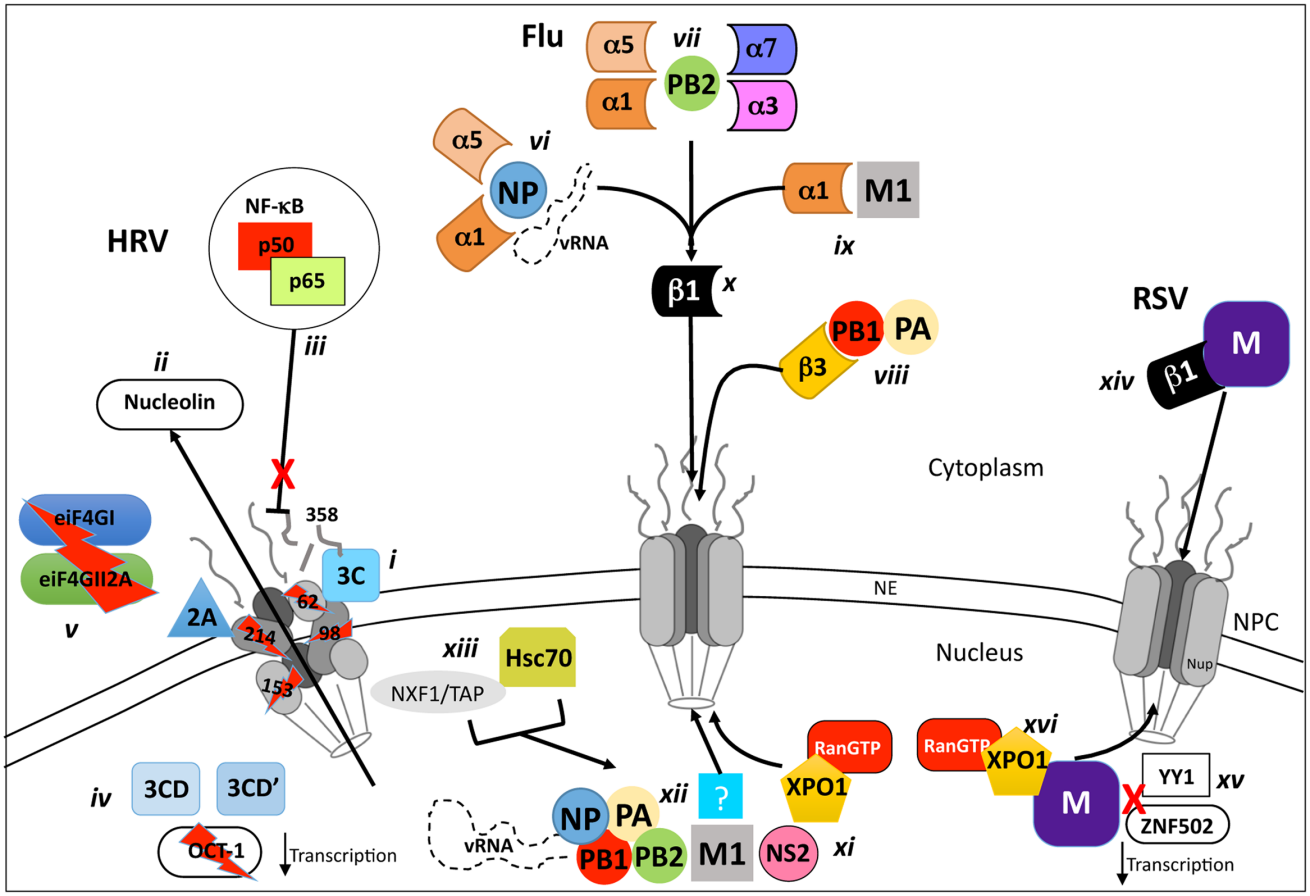
**Schematic representation of VRD modulation and/or exploitation of host nucleocytoplasmic transport processes**. Inhibition and/or utilization of host-cell nucleocytoplasmic transport are key features of infection by Rhinovirus (HRV), Influenza virus and RSV. During HRV infection, the viral proteases 2A and 3C localize to the NPC **(i)** and degrade nups 62, 98, 153, 214, and 358, causing mislocalization of nuclear proteins such as nucleolin **(ii)** and preventing nuclear import of complexes such as the anti-viral NF-κB transcription factor **(iii)**. Host-cell transcription/translation is severely reduced by the NLS of the 3CD and 3CD’ proteases which degrade the general transcription factor OCT-1 **(iv)** in concert with 2A, which also degrades the cytoplasmic translation elongation factors eIF4GI and eIF4GII2A **(v)**. Efficient influenza virus replication requires the transport of the viral genome and proteins required for its replication (PB1, PB2, PA, and NP) to the nucleus where they form a vRNP complex. The vRNA is transported to the nucleus through binding to NP, which is recognized by IMPα1 or α5 **(vi)** in complex with Impβ1 **(x)**, is transported through the NPC, as is PB2 **(vii)**, which is recognized by either IMPα7, α5, α3, or α1 in complex with IMPβ1 **(x)**. The PB1/PA heterodimer is transported to the nucleus by interaction with the IMPβ1 homologue IPO5 **(viii)** which can bind the NPC directly. The M1 protein, critical for the nuclear export of the vRNP complex is imported to the nucleus via IMPα1/β1 **(ix,x)**. The newly synthesized vRNA (part of the vRNP-N1-NS2 complex) is exported from the nucleus by XPO1 interaction with NS2 **(xi).** An unknown exporter **(xii)** that interacts with M1 has been implicated in this process, as have the proteins Hsc70 and NXF1/TAP **(xiii)**, which are postulated to act as cofactors via an undefined mechanism. The RSV M protein relies on interaction with Impβ1 **(xiv)** early during infection to localize to the nucleus where it suppresses host-cell transcription by potentially blocking the activity of transcription factors such as ZNF502 and YY1 **(xv)**. M is exported to the cytoplasm later in infection by XPO1 **(xvi)**, where it is critical for pro-virion assembly.

In recent years, a hallmark of picornavirus, and thus HRV infection, is the shutdown of regulated host-cell nucleocytoplasmic transport (see **Figure 2**), contributing to reduced cellular transcription and translation, although viral transcription/translation continues unabated. The disruption of host-cell nuclear transport has been attributed to the specific proteolysis and degradation of the FG-containing nups 62, 98, and 153 within the NPC by the viral proteases 2A and 3C (Ghildyal et al., 2009b; Park et al., 2010; Watters and Palmenberg, 2011; Walker et al., 2013; see **Figure 2i**), leading to disruption of classical nucleocytoplasmic shuttling (Gustin and Sarnow, 2002; see **Table 1**). The general disruption of nuclear transport can be observed early in HRV infection whereby endogenous nuclear proteins such as the RNA associated La and Sam68 proteins (Itoh et al., 2002; Wolin and Cedervall, 2002) are mislocalised to the cytoplasm, along with the critical ribosome maturation factor, nucleolin (**Figure 2ii**; Gustin and Sarnow, 2002) leading to cell-cycle arrest and subsequent apoptosis (Ugrinova et al., 2007). In an *in vitro* semi-intact cell system, GFP-tagged 3C was found to disrupt both active (IMP-mediated) and passive (size exclusion) nuclear transport through degradation of nups 358, 214, and 153 (Ghildyal et al., 2009b). Interestingly, nup62 was not degraded, implying that proteolysis of specific nups within the NPC may be through the concerted action of 2A and 3C.

**Table 1:** Summary of respiratory virus protein interactions with components of the host nucleocytoplasmic transport system.

Virus	Viral Protein	Host protein	Effect of interaction	Effect of disrupting interaction on virus titre	Reference
Rhinovirus	2A	nup62	Disruption of host nucleocytoplasmic transport	N/A	Gustin and Sarnow (2002)
	2A	nup98			
	2A/3C	nup153			
	2A/3C	nup214/358			
RSV	M	IMPβ1	IMPβ1 transports M to nucleus to initiate host-cell transcriptional inhibition and increase virus production	Mutation of M NLS results in 20-fold reduction in virus titre	Ghildyal et al. (2005a)
	M	XPO1	Nuclear export of M by XPO1 is absolutely essential to initiate virion formation	Mutation of M NES abolishes RSV virus production; inhibition of XPO1 mediated nuclear export using the XPO1 inhibitor LMB reduces RSV titre > 10-fold	Ghildyal et al. (2009a)
Influenza	NP	IMPα1, IMPα5	NP binding to α1 or β5 allows nuclear import of NP through Impβ1	Cells treated with peptides that compete with NP binding for Impα show 2–5 log reduction in Flu virus titre. Granzyme K mediated proteolysis of Impα/β reduced NP nuclear import and subsequent Flu viral titre twofold	O’Neill et al. (1995), Cros et al. (2005)
	PB1	IMPβ3	PB1 (heterodimer with PA) binds to IMPβ3 and is trafficked to the nucleus	Mutation of the IMPβ3 recognised NLS in PB1 results in a 4-log_10_ reduction in virus titre	Hutchinson et al. (2011)
	PB2	IMPα1, α3, α5, α7	PB2 shows preference for IMPβ7 in mammalian cells *in vivo*, but it can bind to IMPα3 and α5	Mutation of PB2 NLS results in 100-fold reduction in virus titre	Resa-Infante et al. (2008), Boivin and Hart (2011), Pumroy et al. (2015)
	PA	IMPβ3	Requires heterodimerization with PB1 to undergo nuclear import by IMPβ3	Mutation of the IMPβ3 recognised NLS in PB1 results in a 4-log_10_ reduction in virus titre	Hutchinson et al. (2011)
	NS2 (NEP)	XPO1	Allows nuclear export of vRNP-M1-NS2 complex from nucleus to cytoplasm where viral budding occurs	The XPO1 inhibitor LMB almost completely suppresses influenza virus levels in infected MDCK cells. XPO1 inhibitor Verdinexor inhibits influenza virus A and B replication in tissue culture and a mouse model.	Watanabe et al. (2008), Brunotte et al. (2014), Gao et al. (2014), Perwitasari et al. (2014)
	M1	IMPα1 (porcine)	M1 shows interaction with porcine IMPα1, but this has yet to be confirmed in human cells	N/A	Liu et al. (2014)
		Exporter (?)	Leucine-rich NES region identified in M1. Mutation causes accumulation of vRNP in the nucleus, even in the presence of NS2	Alanine mutation of the M1 NES reduced Flu viral titre 200–300-fold	Cao et al. (2012)
	vRNP-M1-NS2	Hsc70	Hsc70 has been shown to interact with M1 and may help mediate vRNP nuclear export in the absence of NS2		Watanabe et al. (2014)
		NXF1/TAP	NXF1/TAP is required for the nuclear export of viral mRNA encoding HA, NA, M1, NS1, and M2	siRNA depletion of NXF1 reduced Flu viral titre 100-fold	Read and Digard (2010)

*IMP, importin; nup, nucleoporin; LMB, leptomycin B.*

In addition to its role in nup degradation, 3C in the context of the larger 3CD and 3CD’ precursors appears to localize in the nucleus and degrade the general transcription factor OCT-1 (see **Figure 2iv**; Amineva et al., 2004), leading to a rapid loss of host-cell transcription early in infection (2–4 h). In parallel, the eukaryotic initiation factors (eIFs) eIF4GI and eIF4GII2A, which form part of the eIF4F complex that recognizes capped-RNA, are degraded by 2A (**Figure 2v**) further contributing to halting host-cell translation (Liebig et al., 2002) but not IRES-mediated HRV RNA translation.

During HRV infection, the production of INF-β mRNA through the Type 1 IFN response is attenuated, leading to dampening of the antiviral response (Kotla et al., 2008). Although the precise mechanism leading to the cause of this reduced response has yet to be elucidated, it is entirely plausible that the deregulation of host nucleocytoplasmic transport by the 2A and/or 3C proteases may be responsible, by preventing the nuclear import of activated NF-kB (**Figure 2iii**). Although the precise role(s) and kinetics of 2A and 3C protease-mediated nup degradation remain to be determined *in vivo*, it is clear that disruption and degradation of the NPC and cleavage of essential transcription factors by 3C is central to host cell shutdown, through deregulation of host cell nucleocytoplasmic transport to prevent the infected cell mounting an antiviral response.

## Respiratory Syncytial Virus (RSV)

In a similar fashion to HRV, the paramyxovirus RSV replicates entirely within the cytosol of infected cells. The outer surface of the RSV virion is comprised of a lipid-rich membrane that encompasses the nucleocapsid within which resides the ssRNA viral genome that encodes all 11 viral proteins (Ogra, 2004). Although glycosaminoglycans are utilized in cell culture (Hallak et al., 2000a,b), clinical RSV infection is initiated by binding of the large glycoprotein (G; Levine et al., 1987) on the virion surface to as yet uncharacterized cell-surface receptor(s). A potential candidate in this regard appears to be the nucleolar protein nucleolin, believed to be present on the apical surface of lower respiratory tract epithelia (Tayyari et al., 2011); see also (Holguera et al., 2014). Following attachment, the viral fusion (F) protein causes both viral and cellular membranes to fuse, subsequently releasing the ssRNA containing nucleocapsid core into the host cell cytoplasm and allowing viral transcription/translation to proceed.

A key pathogenic factor and component of RSV is the virally derived Matrix (M) protein, which associates with the nucleocapsid and envelope glycoprotein complexes within the virion, and is believed to be a key driver of virus assembly in the infected cell (Ghildyal et al., 2006, 2009a). In addition to this important role, M is also able to traffic early in infection to the host cell nucleus (**Figure 2xiv**), dependent on interaction with IMPβ1 (Ghildyal et al., 2005a; see **Table 1**). Nuclear M inhibits host cell transcription (**Figure 2xv**; Ghildyal et al., 2003), potentially by targeting transcription factors such as the Zinc finger-containing ZNF501 and ZNF502, or the ubiquitous YY1 (Yin Yang 1; Kipper et al., 2015).

Later in infection, M is ferried to the cytoplasm by XPO1 (**Figure 2xvi**; Ghildyal et al., 2009a), where it localizes to viral inclusion bodies (IBs; Lifland et al., 2012), and functions as an adaptor bringing together newly formed nucleocapsids and envelope glycoproteins F and G (Ghildyal et al., 2002, 2005b, 2006) to effect virus assembly. Although RSV replication and assembly occurs exclusively within the cytoplasm, RSV virus with mutations within M’s IMPβ1-recognized NLS are approximately 20-fold attenuated in terms of virus production (Ghildyal et al., 2009a), indicating that M nuclear import through IMPβ1 is central to RSV infection, and represents a viable target for the development of agents to combat RSV infection. Analogously, M nuclear export through XPO1 is an interesting target based on the fact that RSV mutated in the XPO1-recognized NES of M is not viable, presumably due to the critical requirement for M in the cytoplasm later in infection for RSV virion assembly (Ghildyal et al., 2009a); inhibition of XPO1 using the XPO1 specific inhibitor leptomycin B (LMB) added later in infection reduces RSV virus production 20-fold, underlining the utility of targeting M nuclear export as an approach to inhibit RSV (Ghildyal et al., 2009a).

## Influenza (Flu)

Unlike RSV and HRV, which replicate their viral genomes within the host-cell cytoplasm, Influenza virus must transport its genome, in the form of a RNP complex, into the host-cell nucleus in order for replication to occur. The key components of the RNP are the vRNA binding nucleoprotein (NP; O’Neill et al., 1995) and the vRNA-dependent RNA polymerase (vRdRp), which comprises the three subunits, PB1, PB2 (protein basic 1 and 2) and polymerase acidic (PA). NP-dependent nuclear import of vRNA appears to be through interaction of NP with either IMPα1/β1 or α5/β1 (**Figure 2vi**; O’Neill et al., 1995; Wu et al., 2007). Nuclear import of the PB1/PA heterodimer is mediated through recognition of PB1 by IMPβ3 (IPO5; **Figure 2vii**; Deng et al., 2006; Hutchinson et al., 2011). Finally, PB2 is able to interact with a number of different IMPs, including IMPα7/β1, α1/β1, α3/β1, and α5/β1, all of which can transport it into the nucleus (**Figure 2vii**; Resa-Infante et al., 2008; Boivin and Hart, 2011; Pumroy et al., 2015). Thus, various IMPs are responsible for nuclear import of the various proteins that make up the mature vRNP complex, meaning that there are potentially many candidate NLS:IMP targets for therapeutic intervention early in the viral lifecycle (see **Table 1**).

Once the vRNP is localized within the host-cell nucleus, the vRNA initially undergoes a round of replication resulting in the production of 5′ capped and 3′ poly(A) viral mRNA which is exported to the cytosol utilizing the host-cells mRNA transport machinery to undergo translation to produce new viral proteins. A second round of replication then proceeds, whereby positive sense RNAs are produced to serve as templates for the production of negative sense vRNA genomes, which in turn combine with newly synthesized and nuclear localized NP, PB1, PB2, and PA proteins to form new vRNPs (Josset et al., 2008). These vRNPs undergo nuclear export through the action of the viral NS2 (NEP) and M1 proteins that enter the nucleus either via passive diffusion, or potentially through IMPα1 in the case of M1 (**Figure 2ix**; **Table 1**; Liu et al., 2014), and recruit the host export protein XPO1. XPO1 recognizes the vRNP-M1-NS2 complex (**Figure 2xi**) via 2 NESs on NS2, which helps effect transport of the complex out of the nucleus via the NPC. M1 also appears to contribute to vRNP nuclear export through a NES that would appear to be recognized by an export protein other than XPO1 (**Figure 2xii**; see **Table 1**); mutation of the NES results in vRNPs nuclear accumulation (Cao et al., 2012). Additionally, two recent studies have identified additional cellular proteins as co-factors (**Figure 2xiii**) for vRNP-M1-NS2-XPO1 export (**Table 1**); the heat shock protein Hsc70 (Watanabe et al., 2014), which is believed to play a key role in calcium-/calmodulin-dependent nuclear import of SOX proteins nuclear import (Kaur et al., 2013), and NXF1/TAP (Read and Digard, 2010), integrally involved in the nuclear export of cellular mRNA. These may also represent potential candidates for antiviral intervention. Clearly, the host-cell nucleocytoplasmic transport machinery is absolutely required during multiple stages of the Influenza virus life cycle, with both nuclear import and export of vRNPs presenting targets for potential antiviral intervention (Perwitasari et al., 2014).

## Current Therapeutics

Although there are currently a number of therapeutic and prophylactic approaches to manage HRV, RSV, and Flu, there is an ever-present need for new specific and low toxicity treatments for all three.

A number of different treatment regimens targeting the HRV viral capsid and protease proteins have been trialed in the past (De Palma et al., 2008), but none have had any appreciable effect on HRV disease severity (Jacobs et al., 2013). Vapendavir, a capsid binding small molecular inhibitor is currently undergoing Phase 2b clinical trials, with encouraging preliminary data (Matz, 2013), however, as for previous drugs targeting the viral capsid, which is less highly conserved across HRV strains than the other non-structural proteins, there remains a strong likelihood of selection for viral escape mutants (Thibaut et al., 2012). In contrast, the viral proteases 2A and 3C are highly conserved between HRV serotypes (Tapparel et al., 2007) and represent the most likely candidates for successful therapeutic intervention. The most promising HRV protease inhibitor thus far has been Rupintrivir, which specifically recognizes and irreversibly binds a domain within protease 3C that is highly conserved in all picornavirus species (Binford et al., 2007). Although promising results have been obtained *in vitro* and in early challenge trials, a significant reduction in viral load was not achieved during trials with natural infection due to the requirement for the drug to be taken immediately prior or just after infection (Patick et al., 2005); thus further development in regards to HRV was ceased. Recently the enterovirus 3C protease inhibitor SG85 was shown to effectively inhibit HRV infection, with little evidence for resistance arising under long-term serial passage of virus in a tissue culture system in the presence of the drug (Lacroix et al., 2015). Although few small molecular inhibitors have been developed targeting proteases 3C or 2A (Patick et al., 2005), recent studies show that these may prove be a promising avenue in the future.

There is no vaccine currently available against RSV, with previous approaches having caused increased sensitivity and morbidity of premature babies to RSV (Fulginiti et al., 1969). There are currently two prophylactics available, which are able to be safely administered to individuals identified as high-risk of complications if infected with RSV. The monoclonal antibody Palivizumab targets the RSV F protein, and can be administered to pre-identified high-risk infants such as those with chronic lung/heart disease or premature babies (Chavez-Bueno et al., 2007; American Academy of Pediatrics Committee on Infectious and American Academy of Pediatrics Bronchiolitis Guidelines, 2014). Palivizumab can be administered monthly for a maximum of 5 months to maintain therapeutic levels over winter (Saez-Llorens et al., 1998), but although it has helped reduce hospitalization of “at-risk” individuals, it is unsuitable as a large-scale, community wide prophylactic, due to logistic issues and high cost due to the frequency of dosing required (La Via et al., 2013). A more potent derivative, Motavizumab was initially thought to reduce RSV hospitalizations by 25%, compared to those treated with Palivizumab (Feltes et al., 2011), but a recent study of 118 RSV-infected infants has shown that Motavizumab had no appreciable effect on the duration of hospitalization, severity of illness, or wheezing episodes compared to placebo (Ramilo et al., 2014).

The nucleoside Ribavirin in aerosol form has also been prescribed to treat RSV, but appears to have only a moderate effect in terms of reducing days of hospitalization and recurrent wheezing post RSV infection (Ventre and Randolph, 2007). Ribavirin is also extremely costly (~US $14,000 per treatment) in aerosol form, making it unsuitable for use in impoverished countries (Pelaez et al., 2009). Finally, apart from being carcinogenic and gonadotoxic (Narayana et al., 2005), it appears to be a potential teratogen for women of childbearing age who may come into contact with the drug via secondary exposure (e.g., those caring for children receiving Ribavirin treatment; Krilov, 2002). Clearly, the lack of a vaccine, and limitations of current antiviral prophylactics and therapeutics underline the need for safer, low toxicity and low cost antivirals for RSV.

In contrast to RSV, current options to treat Flu include vaccines and anti-virals. Vaccines, although highly successful, are only able to protect against those viruses that are antigenically close to the vaccine viral reference strain used. Thus, in cases such as the 2009 H1N1 pandemic, vaccine production and lead time was 3–6 months, requiring the use of antiviral agents in the interim (Organization, 2009). The two main classes of anti-influenza virus compounds commercially available are neuraminidase (NA) inhibitors and M2 ion channel blockers. The glycoprotein NA is located within the viral envelope and plays a crucial role in viral particle release from infected cells and prevents virus self-aggregation (Itamura, 1997; Yano et al., 2008; Shtyrya et al., 2009). Historically, NA has been a viable, druggable target due to its high conservation amongst influenza virus strains (Yen et al., 2006), surface accessibility and low rate of mutation compared to other viral proteins, but resistant viral strains have started to appear in the last few years (Garcia et al., 2009).

Influenza virus M2 protein forms a transmembrane pore that helps control the acidity of the virion interior and facilitate viral fusion and release of the genome-containing core. Numerous drugs of the adamantine family have been developed over the last five decades (Davies et al., 1964; Grunert et al., 1965; Tyrrell et al., 1965) that block the action of this pore, although their use is falling out of favor due to the rapid occurrence of resistant viral strains (Leonov et al., 2011). Thus, with use of the two major classes of influenza anti-virals becoming more and more problematic, it is imperative that new, broad-spectrum anti-viral agents be developed.

As indicated above, nucleocytoplasmic transport is integral to the majority of the influenza virus replicative cycle. Previous studies have looked at the effect of attenuating the nuclear transport of various influenza virus proteins as an antiviral approach. Peptides derived from IMPα have been used in a competition approach to reduce the level of nuclear NP, resulting in a dose-dependent reduction in viral titre by decreasing influenza vRNA nuclear import (Cros et al., 2005). In addition, results from a reverse genetics system indicate that mutation of the well-conserved IMPβ3-recognized NLS1 and NLS2 region of PB1 results in severely attenuated/impaired viral replication (Hutchinson et al., 2011). Finally, specific proteolysis of Impα/β1 by Granzyme K in influenza virus-infected A549 cells both impaired NP nuclear import and reduced viral titres by 50% (Zhong et al., 2012), highlighting that the nuclear import of the vRNP can be a viable target for therapeutic intervention.

Exportin1-mediated nuclear export has been identified as critical for efficient influenza virus replication to occur. Influenza virus infected cells treated with LMB, an irreversible inhibitor of XPO1, show a dose-dependent reduction in viral titre, with complete inhibition of viral replication at 10 ng/ml (Watanabe et al., 2008). LMB is an unsuitable drug candidate, however, due to its irreversible binding to XPO1 and associated potential toxicity. Recently a new class of orally available SINE (Lapalombella et al., 2012) have been engineered which form a slowly *reversible* covalent bond with XPO1, minimizing cytotoxicity. The SINE compound Verdinexor has recently been found inhibit the replication of various influenza A and B virus strains in cell culture and reduce lung virus titres and associated disease pathology in a mouse model with minimal cytotoxicity (Perwitasari et al., 2014).

## Screening for Inhibitors

**FIGURE 3:**
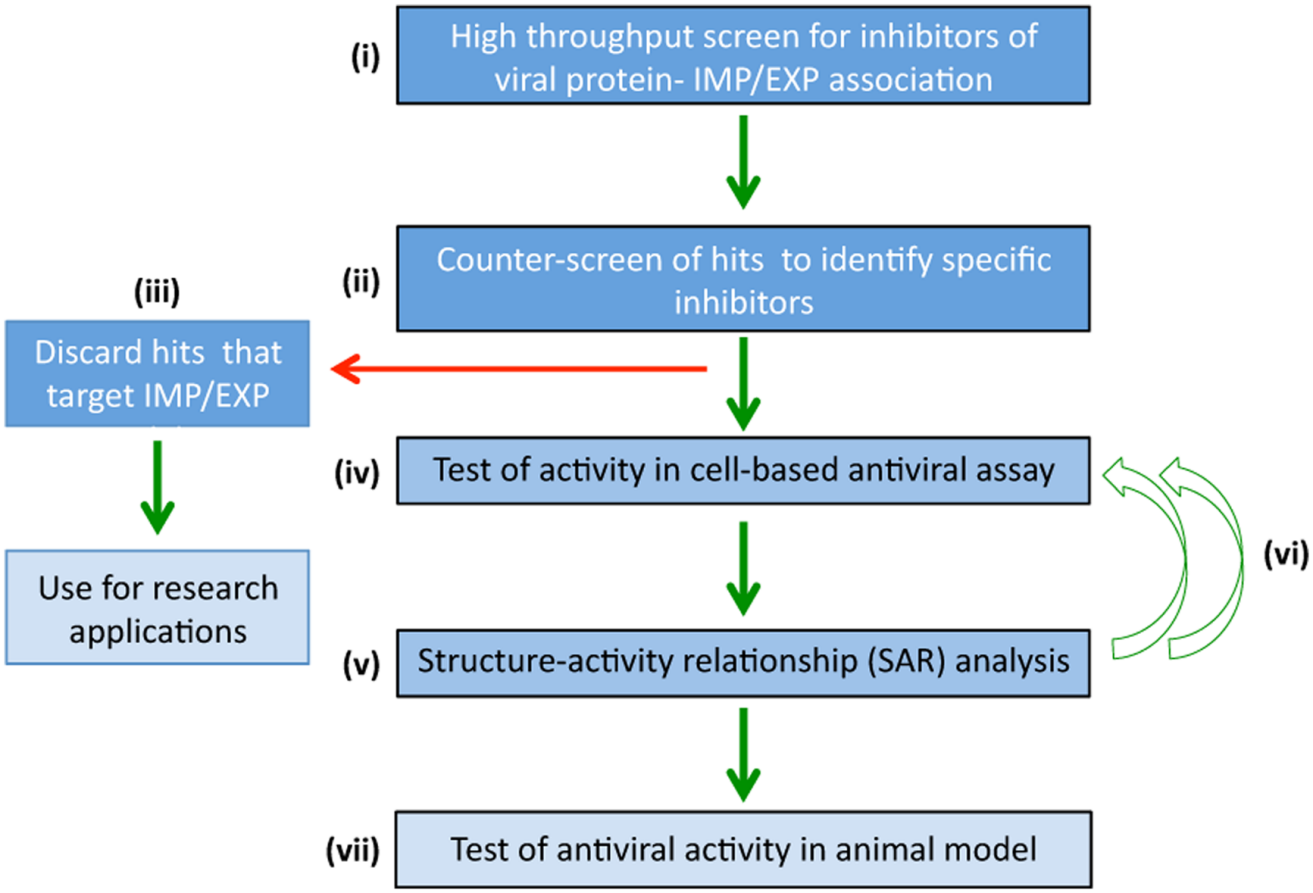
**High-throughput screening to identify specific agents targeting nucleocytoplasmic transport that have antiviral activity.** High-throughput screening strategies to rapidly identify antiviral compounds that specifically block viral:IMP/XPO/host protein interaction. **(i)** Primary screening is performed using a chemical library to identify molecules that inhibit viral protein-IMP/XPO interaction. **(ii)** Hits are counterscreened to identify compounds that target IMP/XPO function directly, which although of interest for research applications, are discarded from further examination in the pipeline toward specific antivirals. **(iii)** Compounds not targeting IMP/XPO function but rather specifically inhibiting viral protein:IMP/XPO interaction are screened in cell-based assays **(iv)** to confirm antiviral activity. **(v)** Structure-activity relationship analysis/focused library screening) is performed to optimize properties of the inhibitor (e.g., pharmacodynamics) in multiple iterations **(vi)**, prior to evaluation of lead compounds in animal models of viral infection **(vii)**.

The main challenge in looking for inhibitors that target ubiquitous systems such as nucleocytoplasmic transport for therapeutic intervention is cytotoxicity. Strategies to overcome this include those where potential hit compounds can be identified that specifically target the interface between viral and host-cell proteins (e.g., IMP-viral protein or XPO-viral protein) rather than the IMP or XPO directly, which would potentially block transport of all host cell proteins that use the IMP/XPO for normal trafficking. High-throughput screening where a counterscreening strategy has been incorporated has proved efficacious in identifying compounds that have proved to be specific inhibitors (Wagstaff et al., 2011; Fraser et al., 2014), with low toxicity and strong antiviral activity (**Figure 3**; Wagstaff et al., 2011, 2012, Fraser et al., 2014). Primary screening (**Figure 3i**) is performed on a compound library to identify molecules that disrupt the interaction between the viral protein-IMP/XPO of interest, followed by specificity counter-screening (**Figure 3iii**) to identify compounds that directly inhibit IMP/XPO function. Only compounds shown to specifically inhibit viral protein-IMP/XPO are selected for further cell based antiviral activity analysis (**Figure 3iv**) and structural refinement (**Figure 3v**; structure/activity determination). Refined molecules are re-screened (**Figure 3vi**) to confirm activity and specificity before evaluation in animal models (**Figure 3viii**). This strategy has been used successfully to identify antivirals targeting nuclear import for HIV and DENV (Wagstaff et al., 2011, 2012; Fraser et al., 2014) – see next section – underlining its intrinsic utility for future endeavors to develop efficacious, non-toxic antivirals.

## Future Prospects

Since many RNA viruses rely on nuclear import of specific viral gene products for efficient replication, nucleocytoplasmic transport of viral proteins represents a viable target for the development of anti-virals, with all of the various interactions listed in **Table 1** thus at least in theory representing potential targets for drug development. Targeting the host cell nuclear import/export machinery itself can clearly have an effect on virus production (Ghildyal et al., 2009a; Prasetyo et al., 2009; Rawlinson et al., 2009; Wagstaff et al., 2012), but it is important to recognize that, in general, targeting host cell proteins directly can lead to toxicity (Newlands et al., 1996),. However, recent work utilizing lower toxicity inhibitors of XPO1 (SINE compounds) against influenza virus (Perwitasari et al., 2014) has progressed into Phase I clinical trails, representing landmark studies in the pursuit of antivirals directed at host nuclear transport components. Drugs targeting viral proteins directly can select for mutations that can reduce binding of anti-viral agents without diminishing viral protein function too drastically (Johnson et al., 2011; Kuritzkes, 2011; Skar et al., 2011; Lundgren and Lazarus, 2012). Targeting the viral protein:host protein interface (Loregian et al., 2002) may be the most profitable in terms of avoiding issues of cytotoxicity, as well as limiting the possibilities for viruses to mutate and still maintain viability. The recognition and subsequent transport of cargo proteins across the NE requires recognition of an NLS by its cognate IMP, with only very minor changes in the NLS tolerated before nuclear transport rates decline (Yang et al., 2010). This makes nucleocytoplasmic transport an attractive therapeutic target, since selective pressure to alter the NLS/NES to prevent drug binding are likely to result in NLSs that are no longer able to be recognized by the host-cell IMP/XPOs and thus fail to mediate efficient nuclear transport.

Mifepristone is an example of a compound that specifically inhibits HIV-1 IN:IMPα/β1 interaction (Wagstaff et al., 2011), and can inhibit HIV infection (Wagstaff et al., 2012), whilst recent work for dengue virus (DENV) shows that a specific inhibitor [*N*-(4-hydroxyphenyl) retinamide] of DENV non-structural protein five nuclear import through IMPα/β1 can protect against infection by all four serotypes of DENV, including severe, antibody-dependent enhanced disease in a lethal mouse model (Fraser et al., 2014). Clearly, targeting the host-pathogen interface in terms of nucleocytoplasmic transport represents an exciting and viable avenue for the future development of novel anti-viral drugs that are likely to be efficacious and importantly, highly specific (Perwitasari et al., 2014).

The approaches of Wagstaff et al. (2011) and Fraser et al. (2014) to derive specific inhibitors targeting the host-pathogen interface used a counterscreening approach (**Figure 3**) to identify all inhibitors that were likely to be directed against IMPs rather than the host-virus interface; only compounds specifically disrupting viral:host protein interaction were pursued, resulting in successful and rapid identification of the inhibitors that ultimately proved to be highly specific for the host-virus interface. This represents an exciting strategy in the future, potentially for HRV 2A/3C nuclear import, for RSV M nuclear import and export, and nuclear import/export of the various influenza virus proteins. Targeting the viral protein-IMP (or viral protein-XPO) interface in the case of these various proteins would appear to be an exciting possibility, with great potential to generate novel, specific antivirals with low toxicity, and little risk of selecting for viral resistance.

## Conflict of Interest Statement

The authors declare that the research was conducted in the absence of any commercial or financial relationships that could be construed as a potential conflict of interest.

## Abbreviations

DAF: decay accelerating factor
Flu: influenza
HRV: human rhinovirus
ICAM-1: intercellular adhesion molecule-1
IFN: interferon
IMP: importin
ISG: interferon stimulated gene
ISRE: interferon stimulated response element
LDLR: low density lipoprotein receptor
LRTI: lower respiratory tract infection
MDA-5: melanoma-differentiation-associated gene 5
NE: nuclear envelope
NES: nuclear export signal
NPC: nuclear pore complex
NLS: nuclear localization signal
Nup: nucleoporin
PAMPs: pathogen associated molecular patterns
RIG-I: retinoic-acid-inducible protein
RSV: respiratory syncytial virus
SINE: selective inhibitors of nuclear export
TLR: toll-like receptors
URTI: upper respiratory tract illness
VRD: viral respiratory disease
vRNA: viral RNA
vRNP: viral ribonucleoprotein complex
XPO: exportin.
